# Morbid Anatomy

**Published:** 1836-01

**Authors:** 


### MORBID ANATOMY.

*On the Anatomical Characters of Cholera.* By W. E. Horner, m.d.

[So much has been written upon the pathology of Cholera within these few years, and to so little purpose, that we are not disposed to pay much attention to any theories or opinions respecting it. We are unfortunately as yet only in that stage of the investigation where the collection and collation of facts, or alleged facts, is all that is permitted to the philosophic enquirer. The time, we conceive, is not yet arrived to justify the attempt to establish any theory of this dreadful malady. In presenting to our readers the following extract, we purposely avoid making any remarks on them further than to state that the observer is a man of honour and practical experience. If it should hereafter be found that he has been mistaken, he will only share the fate of the greatest anatomists who have gone before him; or if he has been led to generalize too speedily from too limited a stock of data, he may console himself in company with the first of living pathologists. We take the facts, or alleged facts, as they are presented to us. Dr. Horner is professor of anatomy in the university of Pennsylvania.]

In Asiatic cholera (says Dr. Horner) the following morbid anatomical characters are found in the alimentary canal:—

1. A copious vesicular eruption, entirely distinct from the tumefaction of villi, muciparous follicles or glands, and which pervades the whole canal.

2. A lining membrane of coagulated lymph, which exists in the small intestines at least, if not in the stomach and colon also, and resembles in texture and mode of adhesion the membrane of croup.

3. Vascular derangements and phenomena, which are confined almost exclusively, if not entirely so, to the venous system.

4. An exfoliation of the epidermic and venous lining of the alimentary canal, whereby the extremities of the venous system are denuded and left pabulous.

[To do full justice to Dr. Horner's opinions, it would be necessary to detail his

* De glandularum intestinalium structura penitiori. Dissert. Inaug. Anatomica, auctore Ludovico Boehm. Berolini, 1835.

views of the anatomical structure of the mucous membrane; but this we cannot do in this place. The following extracts from the second part of his memoir afford some further details respecting the four pathological conditions just stated.]

1. This eruption has been seen by me in four cases, and I would suggest might possibly have been seen in others, had my familiarity with its appearance and means of detection been accurate from the beginning. The form of this eruption is that of a spherical vesicle, commonly from one eightieth to one hundredth of an inch in diameter, with parietes transparent and empty in the dried state, in which alone I have seen it, for the reason that when its parietes are impregnated with a liquid, as water, alcohol, turpentine, or varnish, they are so transparent that they cease to reflect light in an appreciable manner. This vesicle lies upon the surface of what I have designated the superficial venous layer of the digestive canal, perfectly distinct from the follicles, that is to say, having for its base the venous partition between the follicles. In the colon, where the edges of the latter are on the same plain, the vesicles repose as distinctly on the surface of the mucous membrane, as marbles would on a table, and very much after the same manner, one point alone of their circumference resting on the mucous membrane. If it should be permitted me to form a conjecture of the nature of their parietes, I would say that they consisted of the cuticle of the digestive canal. They no doubt contain a fluid in the recent state; but what its character is I have yet to learn, from the difficulty of distinguishing the vesicles themselves at that period. These vesicles in some parts of the jejunum are as thick as they can possibly stand, which, according to the estimate of their size just given, would be, at the rate of some thousands to an inch square, actually six thousand four hundred; but as I have never seen an entire inch square covered in this way, an erroneous impression might be conveyed by stating it as the rule. These vesicles exhibit a decided preference to the roots of the valvulæ conniventes, and are there closely disseminated with scarcely an interval between them; but they decrease in frequency toward the summits of the valvulæ. Their entire number and frequency decline greatly in the ileum and colon, the individual vesicles being much insulated, so as to leave wide spaces between them and others. P. 289.

The pathological appearance which corresponds more than any other with what I have described, is that announced by MM. Serres and Nonat, in the French Lancet for April 1832, under the name of Psorenterie. According to them, it is so little apparent in some subjects, that it would not be perceived without much attention, but very apparent in others; it is found to occupy one half or two thirds of the intestinal canal, beginning at the end of the ileum, where it is always larger and more approximated. On one occasion it was seen in the duodenum, where it had gained the free margin of the valvulæ conniventes. P. 290.

A careful perusal of the above description of the psorentery of MM. Serres and Nonat, will satisfy the reader that the eruption which they describe is different in many particulars from the one announced by myself; that the latter consists of vesicles forming entire spheres, hollow, and much smaller in diameter; and that it may be considered as a specific eruption of cholera heretofore unnoticed. Appended also as it is to the surface of the superficial venous layer, it is never seen in places where the latter has been lost in the progress of the complaint; whereas the psorentery of M. Serres is seen under all circumstances of morbid change noticed by its describers. P. 290.

The precise state of the venous system of the digestive canal is, among all the traits of cholera, that which will most fully account for its destructiveness to human life. The minute anatomy of this system has been explained at page 60 and the following, and we now resume the general fact, that the mucous membrane is formed by an intertexture of these veins, resembling a net, or more exactly a plate of metal pierced with holes; these holes being the follicles, whose aggregate number is forty-six millions at least, and probably much more. When cholera has lasted for a few days, this venous intertexture, which I have denominated for reasons stated, the superficial venous layer, is exfoliated from the stomach, and larger intestines especially, but also in a degree from the small. P. 292.

In regard to the existence of a layer of coagulated lymph on the surface of the digestive canal, in corroboration of my own observations, we have the testimony of Corbyn—for the same being found in the disease as it appeared in India, and of Gerardin and Gaimard—of a similar occurrence in that of Russia. The latter indeed states that the sanguineous afflux, or the active congestion directed upon the intestinal tube, appears to be concentrated chiefly upon the mucous coat of the small intestine. This membrane is swollen, spongy, impregnated with a white fluid; the exudation, of which it is the seat, at first clear and aqueous, takes a more consistent aspect, and forms a lining to it of a flocculent or gelatinous layer, sufficiently like a pseudo-membrane. They add, indeed, what I have never seen, that this layer is sometimes traversed by very fine capillary vessels, which are remarked principally at the points which adhere the most strongly to the membrane of the intestine. There could not be a better evidence than this of the analogy of this layer of fibrine with that of pleurisy or pericarditis, the uniform tendency of which is to become organized by vessels shooting into it. In one specimen which fell under my notice, the adhesion was so strong between the jejunum and this factitious membrane, that I regret not having inspected minutely the part, with the view to test this very question. The case, it will be observed, terminated in eight hours from the invasion, and was attended with a strong inflammatory tinge. The mucous membrane here on being put into spirit of wine, and suspended as a preparation, presented that turgescence in its structure, and villi, and apparent impregnation with a white liquor just spoken of. P. 288.

*American Journ. of the Med. Sciences, May and Aug.* 1835.

---

*On Tubercles.* By J. A. Rochoux, Physician to l'Hospice de la Vieillesse (Hommes). (Hospital for Aged Men.)

M. Rochoux has altered his opinion as to the primary forms of tubercles, from examining them with the microscope, and has written a sensible paper embracing the pathological anatomy, diagnosis, causes, and treatment of phthisis. The only novelty that he brings forward relates to the earliest periods when tubercles can be seen, which is a stage earlier than was admitted by Laennec, who satisfied himself with describing the granular state as the first. M. Rochoux does full justice to the merits of the descriptions given by Bayle and Laennec of the progress of tubercles, from the granular stage to that of softening, but considers that the claim of priority in these pathological discoveries is due to our countryman Stark. M. Rochoux supposes that the granular stage of every tubercle is preceded by a small body of the tenth or twelfth of a line in diameter, brilliant as satin, or mother of pearl, and of all shades between a pearly grey and a slight rosy tint, perfectly homogeneous in its tissue, and without any trace of vessels. The appearance of various sections of these bodies shows that they are wholly solid, and not fluid internally. They are united to the tissue of the organ in which they are developed, by numerous filaments as delicate as the spider's web, yielding to the slightest traction, and the broken extremities of which form around the tubercular point a kind of tomentum like swan's down. This eventually becomes the grey or granular tubercle. This appearance is more marked accordingly as the tubercle is recent, and is therefore most evident in young subjects. This early condition of tubercles has been remarked by M. Rochoux in the lungs, liver, pleura, and peritoneum. *Journal hebdomadaire de Médecine,* Mai, 1835. No. 18—20.

